# Experience in application of modified sling in treatment of Moderate Stress Urinary Incontinence

**DOI:** 10.12669/pjms.36.7.2619

**Published:** 2020

**Authors:** Jing-yang Guo, Feng An, De-qiang Gu, Wenzeng Yang

**Affiliations:** 1 Jing-yang Guo, Department of Urology, Affiliated Hospital of Hebei University, Baoding, Hebei, 071000, P. R. China; 2 Feng An, Department of Urology, Affiliated Hospital of Hebei University, Baoding, Hebei, 071000, P. R. China; 3 De-qiang Gu, Department of Urology, Affiliated Hospital of Hebei University, Baoding, Hebei, 071000, P. R. China; 4 Wenzeng Yang, Department of Urology, Affiliated Hospital of Hebei University, Baoding, Hebei, 071000, P. R. China

**Keywords:** Stress urinary incontinence, Sling modification, Surgery

## Abstract

**Objective:**

To observe the clinical significance of modified sling in the treatment of moderate stress urinary incontinence (SUI).

**Methods:**

From January 2016 to January 2019, eighty patients with moderate urinary incontinence who were hospitalized in our hospital were randomly divided into two groups. 40 patients in the experimental group underwent modified sling transvaginal tension-free mid-urethral suspension. Modification method of the sling: cut the sling to a remaining length of about 6~7cm, properly connect the barbed sutures (V-LOCK) on both sides of the sling, and insert the urinary incontinence sling from the urethra to the obturator membrane, from the obturator membrane to the thigh. The inner skin area is replaced by the V-LOCK line. The 40 patients in the control group were unmodified ordinary slings. The operation time, the local pain of the inner thigh after the operation, and the improvement of postoperative urinary incontinence symptoms were compared and analyzed between the two groups.

**Results:**

Both groups of patients were successfully operated. The operation time was 16.36 minutes in the experimental group and 27.18 minutes in the control group. The difference in operation time between the two groups was statistically significant (p=0.00); the catheter was pulled out on the third day after the operation. One patient in the group had urinary effort, four patients still had urinary incontinence symptoms, the remaining 35 patients had good urinary control (effective rate 87.5%), five patients in the control group still had urinary incontinence, two patients had urinary effort, and the remaining 33 patients had urinary control Good, (effective rate 87.5%), there was no significant difference in surgical effectiveness between the two groups (p=0.53); follow-up for 12 to 36 months, no significant long-term complications occurred, the pain score of the inner thigh of the experimental group was significantly lower than that of the control group ,statistically significant (p=0.04).

**Conclusion:**

The efficacy of the modified sling in the treatment of moderate SUI is the same as that of the traditional sling, but the operation time is shorter, the operation is simpler, and the local pain is significantly reduced.

## INTRODUCTION

Stress urinary incontinence (SUI) is a common disease of middle-aged and old women, especially in women who had vaginal delivery.^1^ The vast majority of new-onset SUI pre-menopausal, and post-menopausal women rarely develop new-onset urinary incontinence.^2^ Except that some mild patients can have a certain therapeutic effect through pelvic floor function exercise.^3^ At present, the most commonly used minimally invasive surgery for SUI is transvaginal tension-free mid-urethral tape.

For mild SUI (type I), single-incision sling or needle-free sling with minimal injury and pain can be used, while for severe SUI (type III), a small number of severe patients require artificial sphincter,^4^ the standard transretropubic tension-free vaginal tape (TVT) is generally used. For moderate SUI (type II), transobturator tension-free tape (TVT-O or TOT) is mostly used. In TVT-O or TOT, the puncture does not pass through the pelvic cavity, so the incidence of intestinal injury and bladder injury reduces significantly. However, due to the sling passing through medial thigh muscles and the puncture points of the skin on bilateral medial thigh in this surgery, pain and limited activities of the medial thigh are the main complications of the patients after surgery. Moreover, single-incision sling has small support strength and some limitations in that the suspension part is only located in the obturator membrane.

In this study, the sling was cut and modified before placement in transobturator tension-free tape (mainly TVT-O) to shorten the length of the sling, and both ends of the sling were fixed with V-LOCK suture. After placing in the body, the modified sling was partially located in the urethra to the obturator membrane, similar to the location of single-incision sling. In addition, V-LOCK suture was used from the obturator membrane upwards to the skin of the medial thigh to enhance traction and support. Postoperative urinary control was good, and the incidence of pain in the medial thigh was low.

## METHODS

### Ethical Approval

The study was approved by the Institutional Ethics Committee of Affiliated Hospital of Hebei University, and written informed consent was obtained from all participants.

### Inclusion Criteria

1. Patients with moderate SUI, 2. patients with high activity of the urethra (Q-tip > 30°),^5^ three. patients with urodynamics indicating urinary leakage in the bladder of 60-90 cmH_2_O.

### Exclusion Criteria

Patients with severe cardiopulmonary diseases which were not corrected by active preoperative preparation, 2. patients with mild and severe SUI, 3. patients with urethral fixation (Q-tip < 10°), 4. patients with prolapse of the bladder or the uterus, 5. patients with bladder neck obstruction, overactive bladder (OAB), and large volume of residual urine (> 100 ml), 6. patients with other types of urinary incontinence, 7. patients with urodynamics indicating urinary leakage in the bladder < 60 cmH_2_O, 8. patients with coagulation dysfunction or near-term oral administration of anticoagulants.

### Data of Patients

From January 2016 to January 2019, eighty patients with moderate SUI were hospitalized in Affiliated Hospital of Hebei University. The patients were randomly divided into two groups, with 40 patients in each group. The observation group received TVT-O using a modified sling, while the control group was treated with traditional TVT-O. In the observation group, the patients aged from 43 to 78 years (mean age, 59.67 years), and had a course of disease of 2-7 years (mean course of disease, 3.50 years). In the control group, the patients aged 42-78 years (mean age, 61.50 years), and had a course of disease of 2-6 years (mean course of disease, 3.42 years). No statistical differences were found in general data between the two groups (Table-I). All the patients in both groups had vaginal delivery but no previous history of surgery for SUI. Preoperative examination suggested positive results in induction test and bladder neck lifting test.

**Table-I:** General data of the two groups (n=40, *X̄*±S)

General data	Observation group	Control group	t/x^2^	p
Age (year)	59.67 ± 9.78	61.50 ± 10.48	0.44	0.66
Proportion of age > 70 years (%)	(2/11)	(3/10)	2.77	0.99
Course of disease (year)	3.50 ± 1.44	3.42 ± 1.11	0.15	0.88
Qmax	24.52±3.91	24.93±3.48	0.49	0.62
Residual urine	7.92±7.25	7.20±5.92	0.48	0.63

*P* > 0.05

### Preoperative preparation

All the patients underwent routine blood and urine test, blood biochemical examination, ultrasound of the urinary system for determining residual urine, gynecological ultrasound for excluding gynecological diseases, vaginal and cervical examination and imaging for excluding external vaginal and cervical diseases, to correct urinary tract infection and cardiopulmonary dysfunction. Patients over 70 years old routinely received local application of the vagina with estrogen ointment for at least one week. All the patients underwent urodynamic examination. In the observation group, Q_max_ was 17.5-28.7 ml [mean, 24.52ml (24.52 ± 3.91)], and the volume of residual urine was 0-20 ml [mean, 7.92 ml (7.92 ± 7.25)]. In the control group, Q_max_ was 19.1-29.7 ml [mean, 24.93 ml (24.93 ± 3.48)], and the volume of residual urine was 0-23 ml [mean, 7.20 ml (7.20 ± 5.92)]. There was no significant difference in residual urine and maximum urine flow rate (Qmax) between the two groups (Table-I). In the two groups, Valsalva’s urodynamic leak point pressure was 60-90 cmH_2_O. The analysis combined with symptoms revealed moderate SUI.

### Surgical Methods

**Fig.1:**
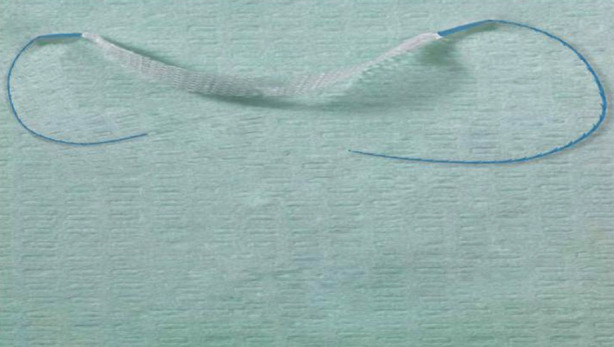
Modified sling (the sling was properly cut short, and firmly connected with barbed suture).

**Fig.2:**
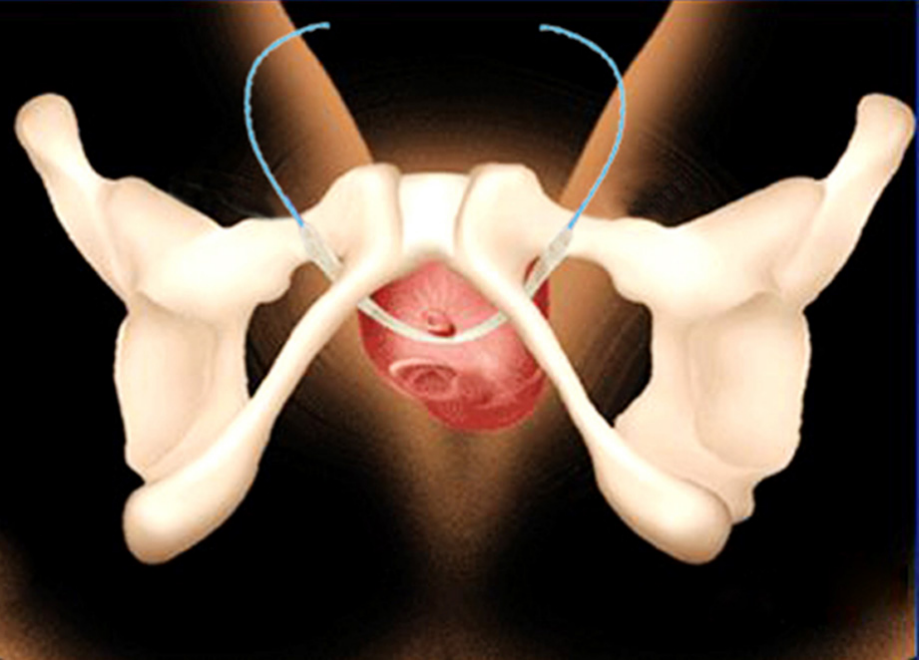
Situation after the placement of modified sling (the urinary incontinence sling was from the urethra to above the obturator membrane, and barbed suture was used from the above obturator membrane to puncturing the skin of the medial thigh through medial thigh muscles).

TVT-O device is equipped with urinary incontinence sling, spiral puncture needle and butterfly-shaped guide. A large-pore polypropylene mesh was used (> 75 um). In the observation group, surgery was carried out using the modified sling. The sling was properly cut short, with the length of about 6-7 cm. The ends of the sling were fixed properly with barbed suture (Fig.1). Under general anesthesia, the patients were in the exaggerated lithotomy position, with the perineum fully exposed. Surgical field and the vagina were disinfected routinely, and urinary catheter was indwelt. Normal saline was injected from the anterior wall of the vagina about 1.5 cm away from the external orifice of the urethra into about 20 cm below the vaginal mucosa, an incision with a length of about 1.5 cm was made, and then the vaginal mucosa was separated until the urethra. In addition, the loose tissue layer on the surface of the urethra was separated to bilateral pubic branches at an angle of 45° with the midline. After satisfactory separation, butterfly-shaped guide was placed through the vaginal incision until the back of the inferior pubic branch, which was guided by fingers in the vagina to avoid damaging the vaginal wall. Barbed suture was inserted into the hole of one side of the spiral puncture needle, and entered the back of the inferior pubic branch closely along the butterfly-shaped guide. After withdrawing the guide, the puncture needle was passed around the pubic branch and out from the clitoris two cm outside of the crumple of ipsilateral thigh root horizontally. Then, the other side was punctured in the same way. Barbed suture puncturing the skin on both sides was stretched to adjust the sling until the urethra was supported without tension, and the space between the urethra and the sling could hold the tip of a rotatable vessel forceps. The excess barbed suture was cut off and the skin incision was glued with biological glue. The vaginal incision was sutured with 2-0 absorbable suture, and the vagina was filled with diluted iodophor gauze. After the surgery, the urinary incontinence sling was around the urethra until passing through the obturator membrane, and V-LOCK suture was used from the obturator membrane to the skin of the medial thigh (Fig.2).

The surgical method of the control group was the same as that of the observation group. During the surgery, the sling was not cut, and directly inserted into the hole of the spiral puncture needle for puncture. Additionally, the sling was led out from the medial thigh, and the tightness of the sling was adjusted. The excess sling was cut off and the skin incision was glued with biological glue. The vaginal incision was sutured using 2-0 absorbable suture, and the vagina was filled with diluted iodophor gauze.

### Evaluation of Surgical Efficacy

Improvement degree of urinary incontinence symptoms: (1) Significantly: After treatment, the symptoms of urinary incontinence disappear completely or basically, and there is no urine overflow under coughing, exercise, etc., and the urine pad test does not exceed 1g; (2) Effective: The symptoms of urinary incontinence after treatment Improved, the urine output of the pad test decreased; (3) Ineffective: failed to meet the aforementioned requirements or deteriorated. Total effective rate = basic cure rate + effective rate.

### Evaluation of local pain after operation

The patients were assessed with the international digital pain assessment scale, with mild 1-3 points, moderate 4-6 points, and severe 7-10 points.

### Statistical Analysis

Statistical analysis was performed using SPSS 20.0. Data were compared by the paired t-test, and rate by the chi-square test between the two groups. *P* < 0.05 was considered as statistically significant.

## RESULTS

All patients had successful stage-I surgery. The operation time of the experimental group was 14-20 minutes, with an average of 16.36 minutes (16.36±1.92). There was no bladder injury during the operation. The catheter was indwelled for 3 days after the operation. After the catheter was pulled out, one patient had trouble urinating, and the ultrasound showed 200ml residual urine. He was given intermittent urinary catheterization and pelvic floor muscle exercises, and normal urination was restored after two weeks. Four cases still had symptoms of urinary incontinence, and the symptoms disappeared after switching to TVT surgery. The remaining 35 patients had good urinary control without urinary incontinence and urine extravasation. The postoperative follow-up period was 12 to 36 months. No obvious long-term complications occurred. The pain score was 1.92±1.75. Among them, there were two cases of moderate pain, one case disappeared 20 days after operation, and one case disappeared 1.5 months after operation. The rest are painless or mild.

Control group: the operation time was 21 ~ 35min (27.18 ± 5.42). There was no bladder injury during the operation. The catheter was indwelling for three days after the operation. Five cases still had urinary incontinence after pulling out the catheter. The symptoms were improved after changing to TVT operation. Two patients had difficulty urinating. The ultrasound examination showed that the residual urine was 170ml and 200ml respectively. The former was given intermittent catheterization and pelvic floor muscle exercise, and returned to normal urination after two weeks. The latter had persistent urination after operation and was suspended under local anesthesia three months later with * cut, after cutting, urinary retention is improved and urine control is good. The other 33 patients had good urine control. The patients were followed up for 12 to 36 months, no significant long-term complications occurred. The pain score was 3.92 ± 2.66. There were significant differences in operation time (t = 6.96, p= 0.00) and postoperative pain score (t = 2.17, p = 0.04) between the two groups. There was no significant difference in the total effective rate between the two groups(χ^2^=0.39,p=0.53)(Table II and III).

**Table-II:** Comparison of surgical duration and pain score between the two groups (n=40, *X̄*±S)

Observation index	Observation group	Control group	t	p
Surgical duration (min)	16.36 ± 1.92	27.18 ± 5.42	6.96	0.00
Pain score	1.92 ± 1.75	3.92 ± 2.66	2.18	0.04

*P* < 0.05

**Table-III:** Comparison of effective rate between experimental group and control group (n=40, *X̄*±S).

	Significantly Effective	Effective	Ineffective	Total Effective rate*
Experimental group (cases %)	27	8	5	87.5%
Control group (cases %)	23	10	7	82.5%
χ^2^				0.39
p				0.53

* p>0.05

## DISCUSSION

SUI is a common disease in middle-aged and old women, especially in those with vaginal delivery. Its incidence is about 30% in the middle-aged and old.^6^ In addition to behavior therapy and pelvic floor muscle exercise,^7^ the most commonly used minimally invasive treatment is transvaginal tension-free mid-urethral tape in clinic. Since reported the first case of transvaginal tension-free mid-urethral tape (TVT) in 1996, TVT has been the gold standard for the treatment of various types of urinary incontinence.^8^ However, because of the long puncture approach, TVT itself has some surgical complications,^9^ such as bladder injury, perforation (2.5%), intestinal injury (3%) and retropubic hematoma (4%). On this basis, many scholars have made a modification, such as TVT-O, TOT, and single-incision sling emerged in recent years.^10^ To a certain extent, the modified surgical methods reduce the occurrence of surgical complications and postoperative pain. However, the modified surgical methods also have some limitations.

At present, the commonly used surgical method is single-incision sling in clinic. In this surgery, the sling is fixed on the internal obturator muscle and the medial obturator membrane without passing through the medial thigh, so the puncture approach is the shortest, postoperative pain is small and recovery is rapid. However, the route of puncture and sling fixation is the shortest and the fixation is the weakest. Therefore, it is suitable for type-I or partial type-II patients. TVT is the standard surgery for the treatment of SUI, and suitable for various types of urinary incontinence. However, its puncture approach is long and passes through the pelvic cavity. Moreover, puncture approach is closely related to the bladder. Thus, the possibility of hematoma or massive hemorrhage caused by injury of the bladder and vascular plexus in the intestinal tube or retropubic space in the pelvic cavity increases. Due to many surgical complications, it is mostly used in the patients after failure of other surgery. Among the patients receiving the first surgery, this surgery is mainly used for patients with severe SUI.

In TVT-O, the needle is punctured into the vaginal incision and out from the medial thigh through the obturator. The puncture direction of TOT is opposite. The needle is inserted from the medial thigh and then punctured out the vaginal incision through the obturator. Although the puncture direction is different, the puncture approach is the same. This approach does not pass through the pelvic cavity, so the chance of injury in the bladder and intestinal tube or retropubic hematoma, is small, Fusco et al. believe that the secondary damage from the inside to outside direction is less^11^ and the route of sling is longer than that of single-incision sling. Therefore, the load-bearing capacity and firmness of sling after surgery are suitable. It can be used for mild and moderate patients, and it is widely used in clinic. However, the approach of perforation passes through the medial thigh muscles, therefore, postoperative pain often occurs in the medial thigh and perineum.^12^

**Fig.3:**
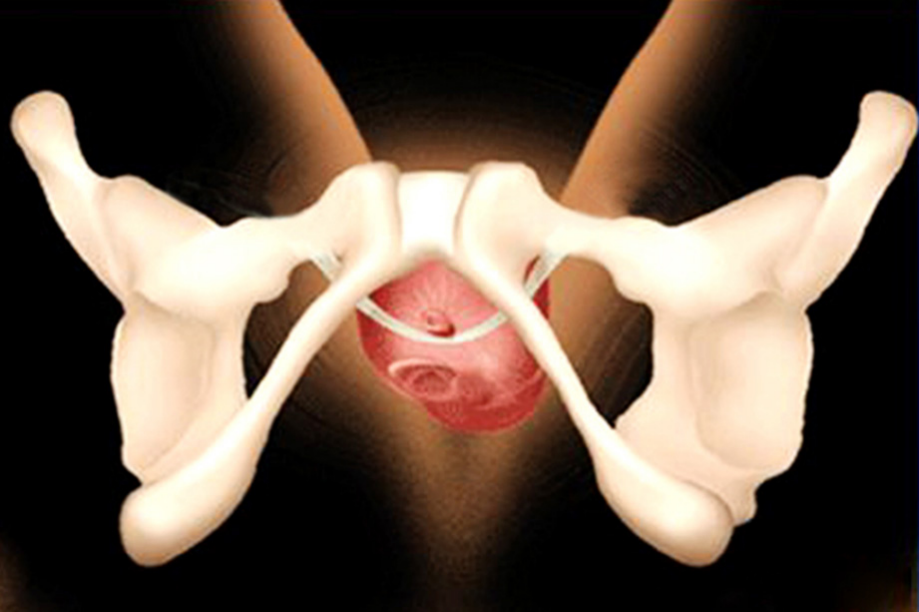
After the barbed suture was absorbed, only the sling was left in the body. At this time, the sling was fixed.

We cut urinary incontinence sling properly and connected it with barbed suture and then indwelt the sling in accordance with the classic TVT-O. After the surgery, the urinary incontinence sling only extended from the periurethra to the obturator membrane and the lateral muscle in the obturator (similar to the position of single-incision sling). In the route from the lateral obturator membrane to the medial thigh puncturing the skin, the sling was replaced with the barbed suture. Its advantage was that the sling could be fixed well and fused with the surrounding tissues within normal time. Moreover, using suture instead of partial sling to pass through the medial thigh muscles reduced muscle stimulation and the incidence of postoperative medial thigh pain. Further, the combination of barbed suture and sling strengthened the position of the sling. The barbed suture could be absorbed within 1-3 months after surgery, during which the sling would be more firm (Fig.3). With the gradual absorption of the barbed suture, the pain in the medial thigh was further relieved. Our study confirmed that the incidence of postoperative pain with the modified sling was lower than that with traditional sling, and the pain was mild, without severe pain. Additionally, the follow-up found that the pain was gradually relieved till disappeared with suture absorption within two months.

The most common cause of recurrence after tension-free mid-urethral tape is the relaxation and displacement of sling.^13^ The authors are used to TVT-O in clinic because of the puncture of TOT from the medial thigh to the vaginal incision. In order to ensure precise puncture and prevent collateral injury, it is necessary to stretch the finger from the vaginal incision to the pubic branch for guidance during the surgery, and a larger vaginal incision is always needed for successful,^14^ so the sling is more likely to displace. In this study, we adopted TVT-O, during which finger guidance was not needed, and the anterior wall of the vagina was incised by only 1.5 cm. The width of the incision was exactly the width of the sling. After placement, the sling was difficult to move. The 24 patients (including the control group) involved in this study were followed up for 12-36 months, all of which involved transvaginal ultrasound to understand the sling position. Except for one patient in the control group whose sling was cut off three months after surgery, the rest patients showed the sling located in the mid--urethra by ultrasound 12 months after surgery. The “knee and foot of the urethra” formed by the sling and the urethra was detected in Valsalva experiment.^15^ Studies have confirmed^16^ that retaining more than one month of sling cutting, there is still a good urine control effect. For some patients who have difficulty urinating immediately after pulling out the urinary catheter after operation, most of them are placed too tightly with sling. Urethral dilation with appropriate strength can achieve obvious effect.^17^

At present, there are many kinds of sling that can be used in clinic. Some of them are hard, always with postoperative complications such as local discomfort in the vagina and coital pain.^18^ Therefore, most centers use urinary incontinence sling with soft texture and good tissue compatibility. After surgery, the vagina is soft locally and comfortable. Additionally, the proportion of retraction is the lowest after the sling is placed into the body. Therefore, it is easy to grasp the tightness during surgery. However, the disadvantage of this sling is small supporting force due to its soft material, so it is not easy to place the sling evenly during surgery. Moreover, the distortion and unevenness of sling will compress the urethra, and thus causing urethral erosion.^19^ Therefore, during puncture, sling indwelling and adjustment of the tightness of sling, the step of keeping the sling even is running through. The modified sling has silk thread structure on both sides, without distortion or curling occurring. Only when adjusting the tightness of the sling, keeping it flat can reach satisfactory placement, and thereby shortening surgical duration. In this study, the mean surgical duration of the observation group was 16 minutes, while that of the control group was 27 minutes, indicating that the modified sling placement had advantage in time. Compared with traditional open surgery, sling surgery has obvious advantages in short-term and medium-term effects and postoperative complications,^20^ but there is no follow-up report on long-term effect.

### Limitations of the study

1. The number of sample cases is small, and the selection criteria are strict. The sample size should be increased and the selection criteria should be reduced to further evaluate the effectiveness of the surgical method; 2. The follow-up time is short and the follow-up time needs to be further extended. Assess its long-term effects; 3. The main reason for the recurrence of urinary incontinence is the change in the position of the sling. The ultrasound understanding of the change in the position of the sling provides an important basis for predicting the recurrence in advance and taking remedial measures. We are also applying it as follow-up content for follow-up In the research, and with the further deepening of the research, the related deficiencies will be gradually improved to provide further evidence-based basis for clinical prognosis and efficacy judgment.

## CONCLUSION

In the treatment of moderate SUI, the modified sling has the advantages of small injury, simple operation, good postoperative urinary control and little complications such as thigh pain.

### Authors’ Contributions:

**JYG**, **FA:** Designed this study and prepared this manuscript, and are responsible and accountable for the accuracy or integrity of the work;

**DQH:** Collected and analyzed clinical data;

**WZY:** Significantly revised this manuscript. All authors read and approved the final manuscript.
